# Sweet Taste Receptor Deficient Mice Have Decreased Adiposity and Increased Bone Mass

**DOI:** 10.1371/journal.pone.0086454

**Published:** 2014-01-22

**Authors:** Becky R. Simon, Brian S. Learman, Sebastian D. Parlee, Erica L. Scheller, Hiroyuki Mori, William P. Cawthorn, Xiaomin Ning, Venkatesh Krishnan, Yanfei L. Ma, Björn Tyrberg, Ormond A. MacDougald

**Affiliations:** 1 Program in Cellular and Molecular Biology, University of Michigan, Ann Arbor, Michigan, United States of America; 2 Molecular & Integrative Physiology, University of Michigan, Ann Arbor, Michigan, United States of America; 3 Department of Internal Medicine, University of Michigan, Ann Arbor, Michigan, United States of America; 4 Musculoskeletal Research, Lilly Research Laboratories, Indianapolis, Indiana, United States of America; 5 Cardiovascular and Metabolic Disease, MedImmune LLC, Gaithersburg, Maryland, United States of America; 6 Diabetes and Obesity Research Center, Sanford-Burnham Medical Research Institute, Orlando, Florida, United States of America; University of Lille Nord de France, France

## Abstract

Functional expression of sweet taste receptors (T1R2 and T1R3) has been reported in numerous metabolic tissues, including the gut, pancreas, and, more recently, in adipose tissue. It has been suggested that sweet taste receptors in these non-gustatory tissues may play a role in systemic energy balance and metabolism. Smaller adipose depots have been reported in T1R3 knockout mice on a high carbohydrate diet, and sweet taste receptors have been reported to regulate adipogenesis *in vitro*. To assess the potential contribution of sweet taste receptors to adipose tissue biology, we investigated the adipose tissue phenotypes of T1R2 and T1R3 knockout mice. Here we provide data to demonstrate that when fed an obesogenic diet, both T1R2 and T1R3 knockout mice have reduced adiposity and smaller adipocytes. Although a mild glucose intolerance was observed with T1R3 deficiency, other metabolic variables analyzed were similar between genotypes. In addition, food intake, respiratory quotient, oxygen consumption, and physical activity were unchanged in T1R2 knockout mice. Although T1R2 deficiency did not affect adipocyte number in peripheral adipose depots, the number of bone marrow adipocytes is significantly reduced in these knockout animals. Finally, we present data demonstrating that T1R2 and T1R3 knockout mice have increased cortical bone mass and trabecular remodeling. This report identifies novel functions for sweet taste receptors in the regulation of adipose and bone biology, and suggests that in these contexts, T1R2 and T1R3 are either dependent on each other for activity or have common independent effects *in vivo*.

## Introduction

Sweet taste perception by the tongue is mediated by the G protein-coupled receptors T1R2 and T1R3 [1,2]. These receptors are reported to function as obligate heterodimers to provide input on the caloric and macronutrient content of ingested food. However, sweet taste receptors have been identified in an increasing number of extra-gustatory tissues [3]–[7], often regulating metabolic processes [8]–[13]. In pancreatic β-cells, sweet taste receptors act to augment glucose-induced insulin secretion in response to artificial sweeteners [11] and fructose [13]. In addition, mice lacking gustducin, a mediator of taste receptor signaling, have reduced glucagon-like peptide-1 (GLP-1) and insulin secretion on account of the loss of sweet taste receptor activity in GLP-1-secreting enteroendocrine cells of the gut [12]. However, sweetener-stimulated GLP-1 secretion appears to be dependent on T1R3, but not T1R2 expression [14], suggesting that these receptors may also function independently of each other in some contexts, perhaps as homodimers.

In addition to effects on insulin and incretin secretion [10], [13], sweet taste receptors may also have metabolic roles in adipose tissue. Masubuchi *et al* reported that T1R2 and T1R3 are expressed in 3T3-L1 cells, and that T1R3 is induced during differentiation and mediates inhibition of adipogenesis by artificial sweeteners [15]. Our group also observed that T1R2 and T1R3 are expressed throughout adipogenesis; however, in our hands, saccharin and acesulfame potassium enhance adipogenesis and suppress adipocyte lipolysis through a mechanism independent of both T1R2 and T1R3 [16]. An additional study has shown that T1R3 knockout (KO) animals are resistant to sucrose-induced obesity and have smaller fat depots on a high-sucrose diet [17], consistent with roles for sweet taste receptors in facilitating adipose tissue expansion. Given that T1R2 and T1R3 are expressed in adipose tissues [16] and have known metabolic functions in other tissues [11]–[14], we performed comprehensive metabolic phenotyping of T1R2 and T1R3 KO mice to further clarify the potential developmental and metabolic roles of sweet taste receptors *in vivo*.

In this report, we investigate the contribution of sweet taste receptors to adipose tissue and metabolic homeostasis by characterizing T1R2 and T1R3 KO mice on a westernized diet. We demonstrate that both KO genotypes show a reduction in adiposity and adipocyte size following this dietary intervention. Despite the reduced adipose tissue expansion in taste receptor KO mice, we detected no or mild changes in glucose tolerance, insulin sensitivity, and energy balance. However, we observed that taste receptor KO reduces adipocyte number in the bone marrow compartment, and increases cortical bone mass and trabecular remodeling. These data represent the first demonstration and comparison of adipose tissue and bone phenotypes in T1R2 and T1R3 KO animals, and this study thereby provides valuable insight into the shared and independent functions of sweet taste receptors *in vivo*.

## Materials and Methods

### Animal Care and Maintenance

T1R2 and T1R3 KO animals were obtained from Björn Tyrberg (Sanford-Burnham Medical Research Institute, Lake Nona, FL), and originally developed by [2] (Charles Zuker; Columbia University, NY). Mice had been backcrossed to 90–95% congenicity with C57BL/6J and were then further backcrossed up to four generations. All experiments were performed on male animals maintained on Western Diet (D12079B, Research Diets, New Brunswick NJ). All mice were housed on a 12-hour light/12-hour dark cycle in the Unit for Laboratory Animal Medicine (ULAM) at the University of Michigan, with free access to food and water. Procedures for this work were approved by the Committee on the Use and Care of Animals at the University of Michigan, with daily care of animals overseen by the Unit for Laboratory Animal Medicine (PRO0001369).

### Animal Measurements

Blood glucose levels were measured with an automated blood glucose reader (Accu-Check, Roche Diagnostics, Indianapolis, IN). Body fat, lean mass, and free fluid were measured in conscious animals using an NMR analyzer (Minispec LF9011, Brucker Optics, Billerica MA) in the phenotyping core of the Nutrition Obesity Research Center at the University of Michigan. Oxygen consumption (VO_2_), carbon dioxide production (VCO_2_), spontaneous motor activity and food intake were measured using the Oxymax Comprehensive Lab Animal Monitoring System (CLAMS, Columbus Instruments; Columbus OH), an integrated open-circuit calorimeter equipped with an optical beam activity-monitoring device. The measurements were carried out continuously for 72 h. During this time, animals were provided free access to food and water through the equipped feeding and drinking devices located inside the chamber. Respiratory quotient (RQ) was calculated as VCO_2_/VO_2_.

### Adipocyte Histomorphometry

Adipocyte size and number were calculated from hematoxylin and eosin stained adipose tissues using MetaMorph Image Analysis software as previously described [18]. Briefly, 100–500 mg of the indicated WAT depots were weighed, placed into 2.5 mL tubes and covered with 10% buffered formalin for at least three days. Prior to paraffin fixation, samples were removed from formalin and placed in 70% ethanol for 48 h. A Leica 2155 rotary paraffin microtome was used to make 5 µm sections at 100 µm intervals across the sample. Samples were then stained and representative photos taken with a Zeiss inverted microscope at 40X objective. After digital processing, adipocyte cross-sectional areas were quantified using MetaMorph Microscopy Automation and Image Analysis software (Molecular Devices, Sunnyvale, CA).

### Glucose Tolerance

Mice were fasted for 16 hours with free access to water. Each mouse was then weighed and glucose measured from tail blood with Accu-Chek Aviva Glucometer (Roche). After obtaining baseline glucose measurements, each mouse was then injected intraperitoneally with a sterile solution of D-glucose at 1 mg/kg. Blood glucose measurements were then taken from tail bleeds for each animal at 15, 30, 60, 90 and 120 minutes.

### Insulin Tolerance

Mice were fasted for 6 hours with free access to water. Each mouse was then weighed and glucose measured from tail blood with Accu-Chek Aviva Glucometer (Roche). After obtaining baseline glucose measurements, each mouse was then injected intraperitoneally with a sterile solution of insulin at 0.75 U/kg. Blood glucose measurements were then taken from tail bleeds for each animal at 15, 30, 60, 90 and 120 minutes.

### Insulin Measurements

Insulin was measured from serum obtained 30 minutes following glucose injection using an ELISA kit (Crystal Chem, Downer’s Grove, IL) and following manufacturer’s instructions.

### Food Intake

Food dispensed into animal cages was initially weighed, and subsequently reweighed every 7 days for 5 weeks. After sifting from animal bedding, collected food wastage was weighed and taken into account when estimating cumulative food consumption.

### Osmium Staining

BM adipocytes were labeled with osmium tetroxide as follows: bones were decalcified for 14 days in 14% EDTA, pH7.4, then washed for 3×10 min in PBS or Sorensen’s Phosphate buffer, pH 7.4, before staining bones in 1% osmium tetroxide for 24 h. Bones were then washed again 3×4–6 h in Sorensen’s Phosphate buffer, pH 7.4, before re-scaning bones with µCT as described below. The tibia/fibula junction was identified and the number of slices between the tibia/fibula junction and the growth plate in the same bone prior to decalcification was determined. This number was subtracted to identify the growth plate on the osmium scan. We then used a threshold of 400 to quantify the marrow fat (excluding any fat in attached tissues) between the growth plate and tibia/fibula junction on the osmium-stained bone. Decalcification, osmium staining and wash steps were done at room temperature.

### µCT Scanning

Mouse specimens were embedded in 1% agarose and scanned using a microCT system. Agarose-embedded mouse bones were placed in a 19 mm diameter tube prior to scanning the length of the bones using the following settings: voxel size 12 µm, medium resolution, 70 kVp, 114 µA, 0.5 mm AL filter, and integration time 500 ms. Density measurements were calibrated to the manufacturer's hydroxyapatite phantom. Analysis was performed using the manufacturer’s evaluation software. Mouse cortical bone was analyzed with a threshold of 280, as follows: 1, the growth plates and tibia/fibula junction were identified and the distance in slices between the two calculated; 2, 70% of this distance was calculated and added to the growth plate landmark; 3, contour at this slice; 4, contour 30 slices up from this initial slice; 4, iterate between these two contours using an outer value of 0 and an inner value of 280, using the stop button to stop. Mouse trabeculae were analyzed using a threshold of 180, as follows: 1, identify the growth plate and go down five slices; 2, draw an internal outline every 10 slices for 50 slices; 3, back-calculate using an outer value of 272 and an inner value of 0. The total volume of mouse bones was determined by contouring around the entire bone between the growth plate and tibia/fibula junction, and then calculating the bone volume and total volume using a threshold of 220.

## Results

### T1R3 KO mice have reduced adiposity on Western Diet

**Figure 1:**
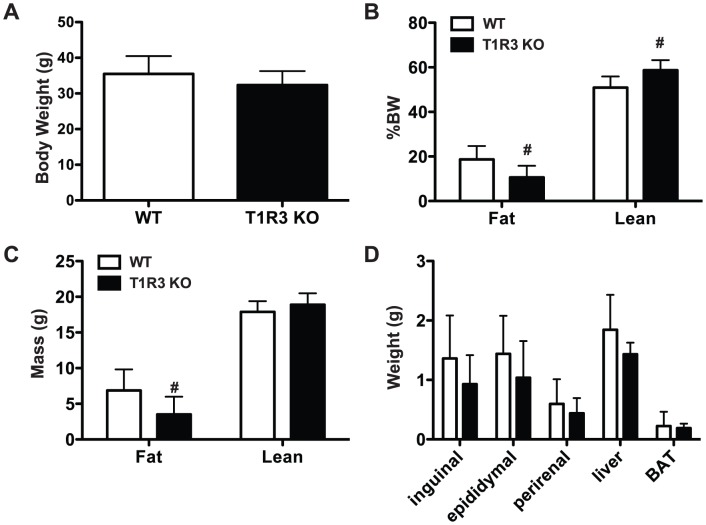
T1R3 KO mice have reduced adiposity on Western Diet. **A) Body weight in WT and T1R3 KO animals after 24 weeks of Western Diet.** Fat mass and lean mass as percent of body weight B) or in absolute mass C) measured by NMR in WT (n = 7) and T1R3 KO (n = 8) animals after 24 weeks Western Diet. D) Weight of inguinal, epididymal, or perirenal fat pads, liver, and brown adipose tissue (BAT) following 24 weeks of Western Diet feeding. In each panel, data are expressed as mean plus S.D. Significance was determined using an unpaired Student’s t-test. *P* <0.05 indicated with #.

Because T1R2/T1R3 function as carbohydrate sensors in the gut and pancreas [12], we hypothesized that sweet taste receptors would serve as mediators of ‘positive’ nutrient signals derived from binding taste receptor ligands in adipose tissue. We therefore anticipated that mice lacking sweet taste receptors might show impairment of adipogenesis or anabolic processes, or have reduced repression of catabolic processes such as lipolysis. Together these deficiencies might lead to metabolic dysfunction. Thus, to assess effects of sweet taste receptor KO on adiposity, we subjected WT and T1R3 KO mice to a 24-week Western diet challenge (41% kcal fat, 43% kcal protein, 17% kcal protein, high sucrose and high cholesterol). At the end of this treatment, body weight did not differ between genotypes (Fig. 1A). However, when the body composition of these animals was evaluated, we observed that T1R3 KO animals had reduced fat mass and increased lean mass as percent of body weight (Fig. 1B). Absolute fat mass was also reduced, though absolute lean mass was not statistically different (Fig. 1C).

To further characterize differences in adiposity in T1R3 KO animals, we measured the weight of individual fat depots (Fig. 1D). However, we observed no differences in the weights of inguinal, epididymal, or perirenal fat depots. There was also no change in liver or brown adipose tissue weight. This disparity between whole body adiposity and weight of individual adipose tissues could be due to greater differences in adipose depots that were not isolated; accumulation of lipid outside of adipose tissue; or result from the amalgamation of small changes within many individual fat pads. The trend towards reduced weight in all KO adipose tissues suggests that the latter might be the case. Taken together, these data are supportive of a role for T1R3 in regulating adiposity *in vivo*.

### Adipose tissue from T1R3 KO mice on Western diet has smaller and more numerous adipocytes

**Figure 2:**
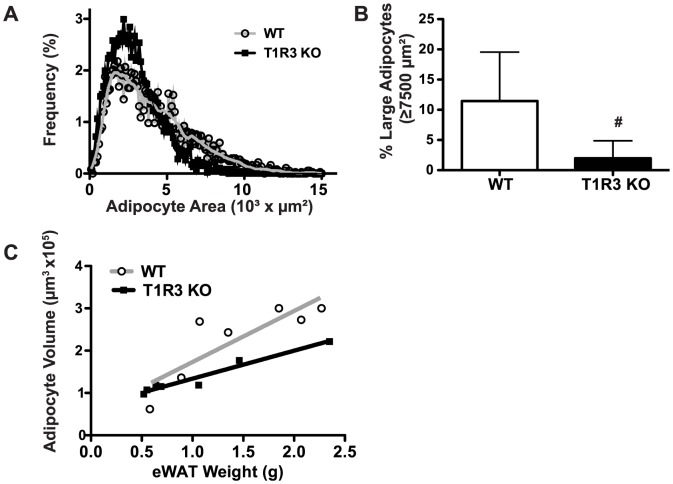
T1R3 KO mice on a Western diet have fewer large adipocytes but increased adipocyte number. A) The frequency distribution reveals that adipocyte sizes in epididymal adipose tissue from WT (n = 7) and T1R3 KO (n = 8) mice on a Western diet are shifted towards smaller cells. B) T1R3 KO animals have decreased frequency of large adipocytes, defined as having a surface area greater than 7500 µm^2^, relative to WT animals. Significance was determined using Student’s t-test. *P* <0.05 indicated with #. Data are expressed as mean plus S.D. C) Linear relationship between eWAT (epididymal white adipose tissue) weight and average adipocyte volume for WT (r^2^  =  0.71) and T1R3 KO (r^2^  =  0.94) mice. Slope (*P*  =  0.14) and intercept (*P* < 0.05).

Reduced adiposity in T1R3 KO animals could be due to decreased adipocyte number, smaller adipocyte size, or both. To determine if T1R3 is a regulator of hyperplasia or hypertrophy we measured the cross-sectional areas of adipocytes in epididymal adipose tissue of WT and T1R3 KO mice. We found that the T1R3 KO animals had a shift towards smaller adipocytes (Fig. 2A), and thus the proportion of large adipocytes was significantly reduced (Fig. 2B). To further explore this observation and estimate relative adipocyte numbers between genotypes, we correlated average adipocyte volume with adipose depot weight for each animal. A weak trend towards a flatter slope, with intercepts statistically different (Fig. 2C), suggests adipose depots from T1R3 mice are characterized by smaller and slightly more numerous adipocytes, especially in those animals with larger adipose depots.

### T1R3 KO mice have mild reductions in glucose sensitivity

**Figure 3:**
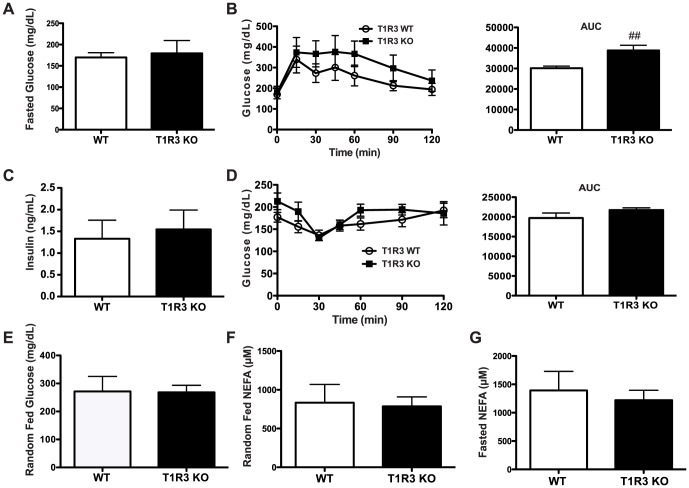
Glucose tolerance is mildly impaired and insulin sensitivity is similar between WT and T1R3 KO mice. A) Fasted glucose of WT (n = 6) and T1R3 KO (n = 9) mice. B) IP GTT in WT (n = 6) and T1R3 KO (n = 9) mice on Western Diet for 23 weeks. Time course following glucose injection (left panel) and AUC (right panel). C) Serum insulin 30 min following glucose injection during IP GTT. D) Intraperitoneal insulin tolerance test (left panel) in WT (n = 6) and T1R3 KO (n = 8) mice on Western diet for 23.5 weeks. Quantification of AUC (right panel). E) Random fed glucose from WT (n = 6) and T1R3 KO (n = 9) mice on Western Diet for 24 weeks. F. Random fed NEFA from WT (n = 6) and T1R3 KO (n = 9) mice on Western Diet for 24 weeks. G. Serum NEFA from WT (n = 6) and T1R3 KO (n = 9) mice on Western Diet for 23 weeks, fasted for 16 h. For each panel, data are expressed as mean plus S.D. Significance was determined using an unpaired Student’s t-test. P <0.01 indicated with ##.

Given the smaller adipocytes present in T1R3 KO mice, we hypothesized that these animals might have altered glucose tolerance. To test this, we first measured blood glucose from WT and T1R3 KO animals that had been fasted for 16 h (Fig 3A). However, we observed no difference between genotypes. We next evaluated the glucose tolerance of T1R3 KO animals by performing intraperitoneal glucose tolerance tests (IP GTT) in WT and T1R3 KO mice after 24 weeks of Western diet (Fig 3B). We observed no significant differences between WT and T1R3 KO mice at any time point following glucose injection (Fig. 3B left panel); however, the area under the curve (AUC) of GTTs from T1R3 KO animals was significantly greater due to small changes in glucose excursion at each time point (Fig. 3B, right panel). Thus, T1R3 KO mice may be mildly glucose intolerant, although fasting glucose was not different between genotypes (Fig. 3A). Elevated blood glucose concentrations are not due to reduced insulin secretion in response to glucose load, as serum insulin did not differ between genotypes 30 min following glucose injection (Fig 3C). To determine if insulin sensitivity was reduced in T1R3 KO animals, we next performed an intraperitoneal insulin tolerance test (ITT, Fig 3D). However, these animals showed no difference in this measure of insulin sensitivity at individual time points (left panel) or in AUC (right panel). Consistent with the mild change in glucose sensitivity observed in T1R3 KO animals, random fed glucose concentrations were also not different (Fig 3E). Given the smaller adipocytes present in T1R3 KO mice, we next hypothesized that these animals might have elevated rates of lipolysis. To test this, we measured fed and fasted serum NEFA concentrations in WT and T1R3 KO animals; however, we did not observe alterations in serum NEFA in either random fed animals (Fig 3F) or those fasted for 16 h (Fig 3F). Serum glycerol concentrations were also similar between genotypes (unpublished data).

### T1R2 KO mice have reduced adiposity on Western Diet

**Figure 4:**
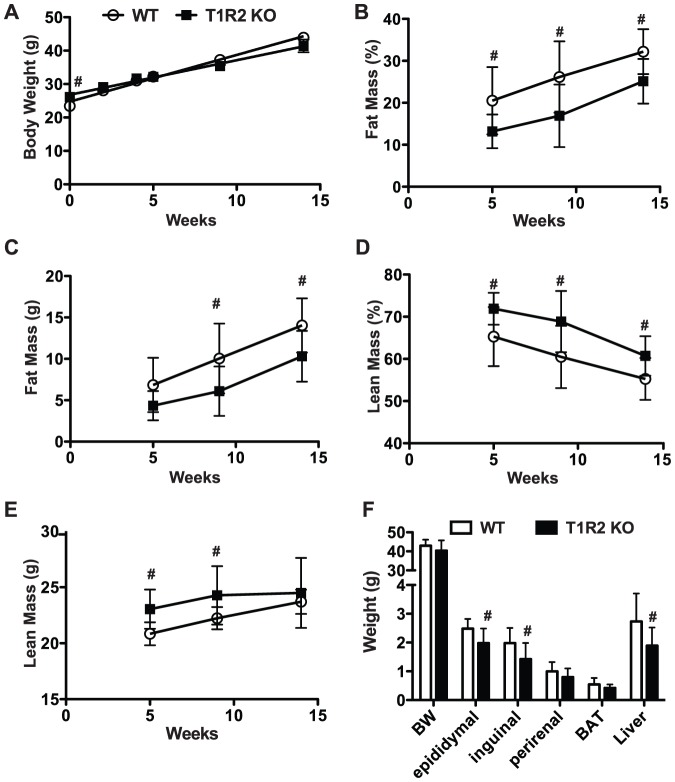
T1R2 KO mice have reduced adiposity on Western Diet. A) Body weight in WT (n = 9) and T1R2 KO (n = 9) mice on Western Diet for 14 weeks. B) Fat as percent of body weight or C) as mass. D) Lean mass as percent of body weight or E) as mass in WT and T1R2 KO animals. F) Adipose depot and liver weights following Western diet feeding. Data are expressed as mean and S.D. Significance was determined using an unpaired Student’s t-test. *P* <0.05 indicated with #.

We continued our investigation of sweet taste receptor functions in adipose tissue by examining the adiposity of T1R2 KO mice upon a Western diet challenge. As T1R2 and T1R3 are generally believed to be obligate heterodimers [1], [2] with both receptors required for function, we predicted that T1R2 KO animals would mirror the phenotypes of T1R3 deficiency. We therefore placed adult T1R2 KO animals on Western diet and evaluated effects on body composition. T1R2 KO animals were heavier than WT controls at the start of the experiment, but after 14 weeks of Western diet there was no significant difference in body weight (Fig. 4A). However, both relative and absolute fat mass were reduced in T1R2 KO mice after 5 weeks of dietary challenge, and this persisted for the remainder of the experiment (Fig. 4B, C). Likewise, lean mass was also increased (Fig. 4D, E). Examining individual adipose depots to further characterize loss of adiposity, we observed that several adipose tissue depots were significantly lighter following the Western diet (Fig. 4F). These data are consistent with the leaner body composition observed in T1R3 KO animals, thus supporting a model whereby the T1R2/T1R3 heterodimer regulates adiposity.

### T1R2 KO mice have smaller adipocytes but equal adipocyte numbers

**Figure 5:**
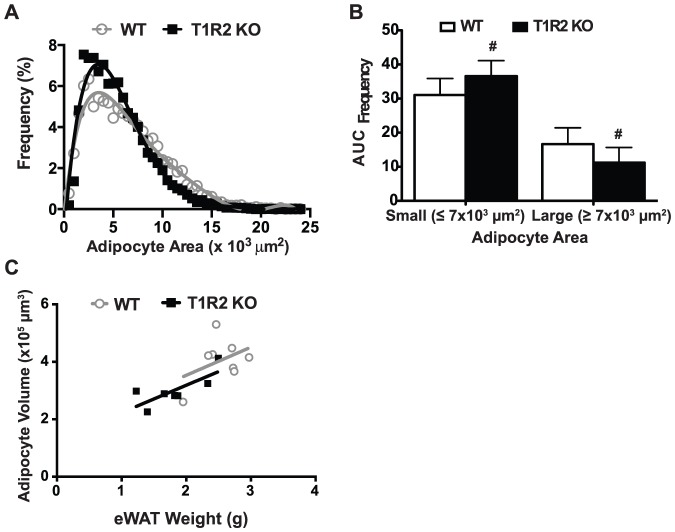
T1R2 KO mice have smaller adipocytes but equal adipocyte numbers. A) Adipocyte size frequency distribution in WT (n = 9) and T1R2 KO (n = 9) mice on Western Diet for 14 weeks shows a greater prevalence of smaller adipocytes in T1R2 KO animals. B) T1R2 KO animals show a decreased frequency of large adipocytes, defined as having a surface area greater than 7000 µm^2^, and increased frequency of small adipocytes, defined as having a surface area smaller than 7000 µm^2^. Significance was determined using Student’s t-test. *P* <0.05 indicated with #. Data are expressed as mean plus S.D. C) Linear relationship between eWAT (epididymal white adipose tissue) weight and average adipocyte volume for WT (r^2^ = 0.16) and T1R2 KO (r^2^ = 0.60) mice. Slope (*P* = 0.98) and intercept (*P* = 0.43)

As the reduced adiposity in T1R2 KO mice mirrored that of T1R3 KO animals, we investigated whether adipocyte size was similarly altered between genotypes. Frequency analysis of adipocyte size in WT and T1R2 KO epididymal fat depots revealed a shift towards smaller adipocytes (Fig. 5A), as observed in T1R3 KO animals. Indeed, T1R2 KO animals had a significantly greater proportion of small adipocytes and a significantly lower proportion of large adipocytes (Fig. 5B). Finally, we again estimated relative adipocyte number between genotypes by correlating average adipocyte volume with fat depot weight using linear regression (Fig. 5C). In T1R2 KO adipose, there was no difference between slopes or intercepts in these correlations, suggesting that reduction in adipose tissue weight is accounted for by smaller adipocytes, rather than fewer adipocytes. Accordingly, the phenocopying of reduced adipocyte size between T1R2 and T1R3 KO mice supports a role for a heterodimeric T1R2/T1R3 sweet taste receptor regulating adipose tissue biology.

### T1R2 KO mice have no changes in glucose sensitivity

**Figure 6:**
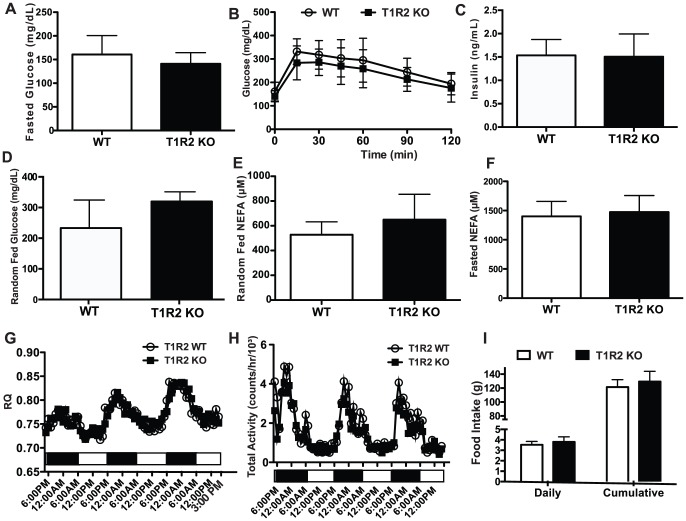
T1R2 KO mice do not have detectable changes in energy balance or glucose homeostasis. A) Blood glucose from WT (n = 9) and T1R2 KO (n = 9) animals on Western Diet for 10 weeks following a 16 h fast. B) IP GTT in WT (n = 9) and T1R2 KO (n = 9) mice following 10 weeks on Western Diet C) Serum insulin in WT (n = 9) and T1R2 KO (n = 9) animals 30 min following glucose injections of IP GTT. D) Random fed glucose from WT (n = 9) and T1R2 KO (n = 9) animals following 14 weeks on Western Diet. E) Non-esterified fatty acids measured from serum of WT (n = 9) and T1R2 KO (n = 9) mice following 14 weeks on Western Diet. F) Non-esterified fatty acids measured from serum of WT (n = 9) and T1R2 KO (n = 9) mice on Western Diet for 10 weeks following a 16 h fast. G) Respiratory Quotient (RQ) and H) total activity were measured on WT (n = 9) and T1R2 KO (n = 9) mice in Comprehensive Laboratory Animal Monitoring System (CLAMS) cages after 3 weeks on Western Diet. I) Food intake in WT (n = 9) and T1R2 KO (n = 9) animals on Western Diet measured over 5 weeks. Data are expressed as mean plus S.D

As KO of T1R2 results in smaller fat depots and smaller adipocytes, we hypothesized that T1R2 KO animals, like T1R3 KO, may have impaired glucose tolerance. We therefore measured blood glucose in WT and T1R2 KO animals following a 16 h fast (Fig 6A), but observed no difference between genotypes. We next evaluated glucose tolerance by performing an IP GTT; however, there was no difference in blood glucose at individual time points (Fig 6B), AUC (unpublished data), or serum insulin 30 min after glucose injection (Fig 6C). Further, we also observed no differences in random fed glucose (Fig 6D). We next measured circulating NEFA concentrations, as reduced adipocyte size could reflect increased lipolysis. However, as with T1R3 KO (Fig. 3A), T1R2 KOs showed no difference in serum NEFA under random-fed conditions (Fig. 6E) or 16 h fasted conditions (Fig 6F).

To investigate other potential mechanisms for reduced adiposity in T1R2 KO mice, we measured energy balance, carbohydrate and lipid utilization, and physical activity in T1R2 KO animals. However, after phenotyping mice with the Comprehensive Animal Monitoring System (CLAMS), we observed no differences in respiratory quotient (Fig. 6G), physical activity (Fig. 6H), or rate of oxygen consumption (unpublished data). Although neither daily nor cumulative food intake differed (Fig. 6I), serum concentrations of the adipocyte-derived hormone, adiponectin, were slightly reduced in serum of T1R2 KO mice (unpublished data).

### T1R2 KO mice have fewer bone marrow adipocytes

**Figure 7:**
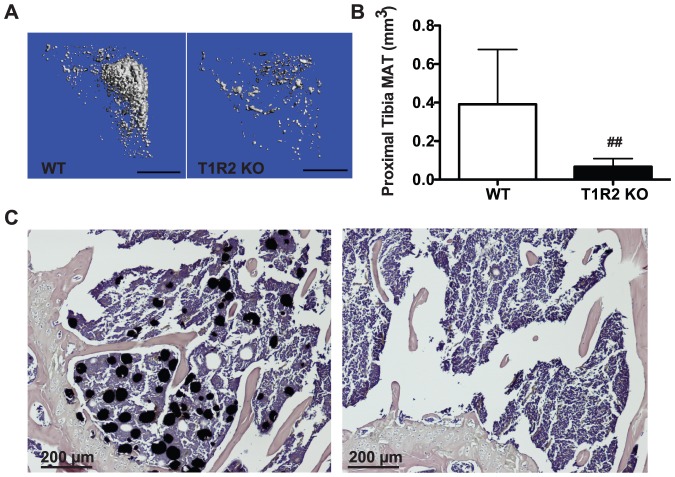
T1R2 KO mice have fewer bone marrow adipocytes. A) Adult WT (left panel) and T1R2 KO (right panel) mice were placed on Western Diet for 14 wks, and bone marrow adipocytes were stained with osmium tetroxide before µCT scanning. Representative µCT shown, WT n = 9, T1R2 KO n = 9. B) Quantification of osmium tetroxide staining in tibia marrow adipose tissue from WT and T1R2 KO mice on Western Diet for 14 weeks. Data are expressed as mean plus S.D. C) Representative osmium tetroxide histology of bone marrow adipocytes in the proximal tibia. WT left panel, T1R2 KO right panel. Significance was determined using an unpaired Student’s t-test. *P* <0.01 indicated with ##.

Lack of T1R2 or T1R3 results in reduced adiposity and smaller adipocytes in peripheral adipose tissue depots. However, these are not the only metabolically important adipocyte populations; bone marrow adipocytes are emerging as an increasingly important regulator of metabolism [19], [20]. We therefore examined adipocyte populations in the bone marrow cavity of T1R2 KO mice by osmium tetroxide staining [21]–[23]. Interestingly, µCT scans of osmium-stained tibiae showed a reduction in the number of bone marrow adipocytes in KO animals (Fig. 7A). Quantification of this staining confirmed that adipose tissue volume in bone marrow of proximal tibiae was significantly reduced in T1R2 KO animals (Fig. 7B). This was further verified by histological analysis, in which fewer bone marrow adipocytes were observed in osmium-stained sections of T1R2 KO tibiae (Fig. 7C). These observations suggest that lack of T1R2 may impair adipogenesis in the bone marrow microenvironment.

### T1R3 KO animals trend towards fewer bone marrow adipocytes

To comprehensively assess the contribution of taste receptors to adipocyte number in the bone marrow compartment, we also quantified bone marrow adipocytes in T1R3 KO animals. Here we observed a trend towards reduced numbers of adipocytes in the media tibia (P = 0.17), but this effect was not significant (unpublished data). These results suggest a stronger regulatory role for T1R2 than T1R3 for bone marrow adipogenesis.

### T1R2 KO animals have increased trabecular bone mineral content and cortical area

**Figure 8:**
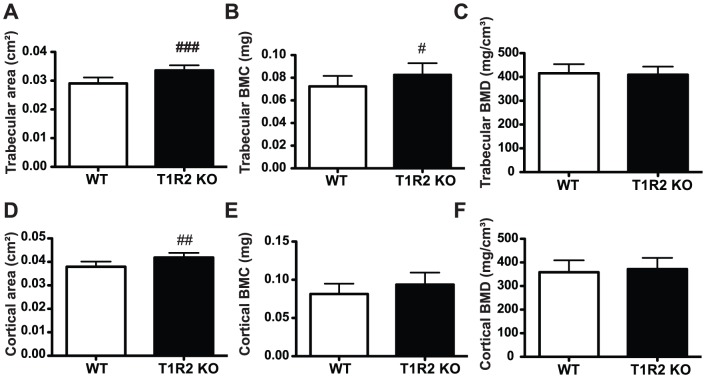
T1R2 KO animals have increased trabecular bone mineral content and cortical area. WT (n = 9) and T1R2 KO (n = 9) mice were placed on Western Diet for 14 weeks, as in Fig 5. Trabecular (A,B,C) and cortical (D,E,F) parameters in tibia were evaluated by µCT. Significance was determined using an unpaired Student’s t-test. *P* <0.05 indicated with #, *P-*<0.01 indicated with ##, and *P* <0.005 indicated with ###. Data are expressed as mean plus S.D.

Our data indicate that loss of T1R2 and T1R3 in the bone marrow cavity may inhibit adipogenesis. As osteogenesis and adipogenesis are often reciprocally regulated [24]–[28], we evaluated the bone mass of these animals to determine if osteogenesis was increased. We performed µCT scans of WT and T1R2 KO mouse tibiae to assess the quantity and architecture of cortical and trabecular bone. We observed that T1R2 KO animals on Western diet had increased trabecular area (Fig. 8A) and trabecular bone mineral content (BMC, Fig. 8B). This combination results in very little change in trabecular bone mineral density (BMD, Fig. 8C), as greater BMC is diffused over the larger trabecular area. Differences were not observed in BV/TV, trabecular thickness, number, or separation (unpublished data). We also observed alterations in cortical bone; T1R2 KO animals showed an increase in cortical area (Fig. 8D), but no change in cortical BMC (Fig. 8E) or BMD (Fig. 8F). Taken together, these results suggest that in bone, T1R2 activation may repress osteogenesis and stimulate adipogenesis.

### T1R3 KO animals have increased cortical area and trabecular remodeling

**Figure 9:**
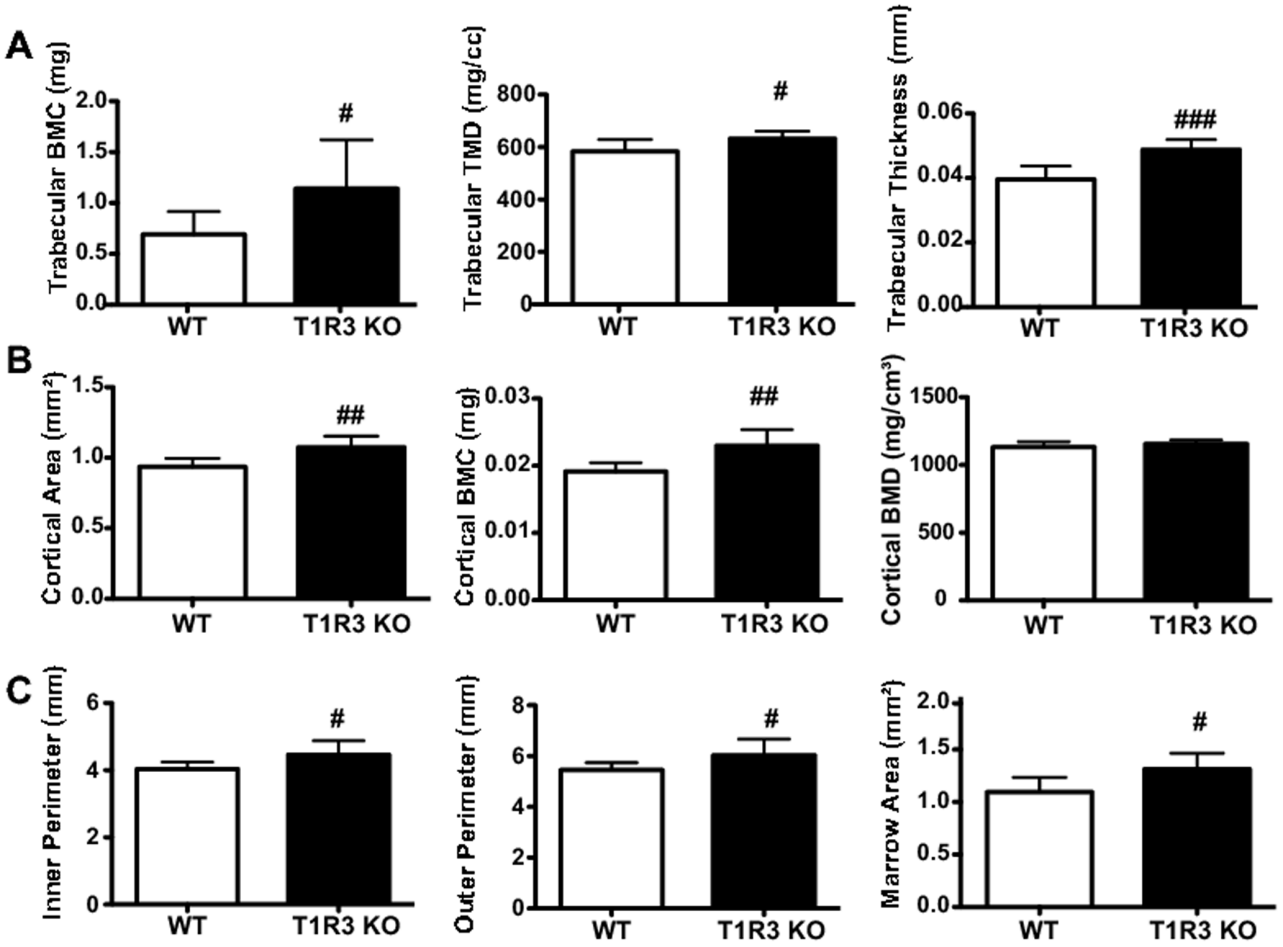
T1R3 KO animals demonstrate trabecular remodeling and increased cortical bone. WT (n = 7) and T1R3 KO (n = 8) mice were placed on Western Diet for 24 weeks. Trabecular (A) and cortical (B,C) parameters were evaluated in the femur by µCT. Significance was determined using an unpaired Student’s t-test. *P* <0.05 indicated with #, *P* <0.01 indicated with ##, and *P* <0.005 indicated with ###. Data are expressed as mean plus S.D.

To determine if effects of T1R2 deficiency on bone mass were also observed in T1R3 KO mice, we independently evaluated femurs of T1R3 KO mice on Western Diet for 24 weeks. T1R3 KO produced several pronounced changes in trabecular bone. Trabecular BMD and tissue mineral density (TMD) were both increased, as was the thickness of individual trabeculae (Fig. 9A), although bone volume fraction and other variables were unchanged (unpublished data). We also observed several significant differences within cortical bone (Fig. 9B). Cortical area (left panel) and cortical BMC (middle panel) were both increased, with cortical BMD (right panel) unchanged. This increase in cortical bone was reflected in the larger inner and outer cortical perimeter in T1R3 KO mice (Fig. 9C, left and middle panel), and a larger marrow area (Fig. 9C, right panel). These data, taken together with increased bone mass in T1R2 KO animals, suggest that sweet taste receptors may have a previously uncharacterized role in the development and/or maintenance of adipose tissues and bone.

## Discussion

In this manuscript, we present results demonstrating reduced adiposity and smaller adipocytes in T1R3 and T1R2 KO animals on Western diet. In support of a previously published report [14], we also observed glucose intolerance in T1R3 KO mice compared to controls. Although we not observe altered glucose homeostasis in T1R2 KO mice on a western diet, the shorter duration of dietary treatment in T1R2 KO animals (10 weeks for T1R2 versus 23 weeks for T1R3) may have contributed to this lack of effect. We found no differences in any other measured metabolic parameters, including circulating NEFA or random-fed glucose. Surprisingly, we also observed that T1R2 and T1R3 KO mice have increased bone mass, which is to our knowledge the first report of sweet taste receptors having a role in bone or bone marrow biology.

The presence of an adipose tissue phenotype in T1R2 and T1R3 KO animals suggests that these receptors may be involved in adipose biology *in vivo*. While the mechanism for reduced adiposity and smaller adipocytes remains unclear, we can speculate on the driving forces behind this phenotype. Smaller adipocytes could be driven by alterations in lipolysis or lipid storage, though circulating fasted NEFA is unchanged (Fig. 3G, 6F); however, this could be a subtle effect overcome by NEFA uptake into liver and muscle. Sweet taste receptor ligands have also been reported to suppress lipolysis [16], which would be consistent with enhanced lipolytic activity in a taste receptor KO model. However, these lipolytic effects *in vitro* are independent of sweet taste receptor activity [16]. It is also unlikely that smaller adipose depots are due to impaired adipogenesis, as adipocyte number in epididymal fat depots was similar or even increased between genotypes. In the absence of changes in food intake, oxygen consumption, or physical activity, these results allow speculation that sweet taste receptors could impact upon lipid utilization, adipocyte expansion, or hormone secretion, and thereby promote reduced adiposity. However, in the absence of adipose tissue-specific sweet taste receptor KO animals, it remains possible that sweet taste receptors impact adipose biology by acting in other tissues, such as the gut or pancreas [11], .

While both T1R2 and T1R3 KO animals have significantly impaired sweet taste sensitivity, there has been some suggestion that T1R2 and T1R3 may be capable of functioning independently as homodimers [14], [15], [29]. Work from the Munger group has suggested that T1R3, in particular, may be the primary mediator of T1R2/T1R3 signaling [14], [30]. The Shibata group has corroborated this idea by suggesting that artificial sweeteners regulate adipogenesis through T1R3, but not T1R2 [15]. However this work is in contrast to our own data, which showed that effects of sweeteners on adipocyte differentiation and lipolysis are independent of T1R2 and/or T1R3 [16]. Our observations here that the loss of T1R2 largely phenocopies the loss of T1R3 suggests that the absence of either receptor is sufficient to impair adipose tissue expansion. In particular, our observation that T1R2 KO animals have more extreme phenotypes in some cases, such as in the loss of bone marrow adipocytes, suggests that the dominant T1R3 activity observed by some groups is not a ubiquitous phenomenon. However, metabolic phenotyping of T1R2/T1R3 double KO animals will need to be performed to definitively address this possibility.

Reciprocal regulation of bone mass and bone marrow adipocytes is an interesting finding of this study. While we cannot interpret the loss of bone marrow adipocytes in T1R2 KO animals as a failure of adipogenesis *per se*, the concurrent increase in bone mass suggests taste receptor involvement in an osteogenesis-adipogenesis developmental axis. There are currently few connections in the literature between taste receptors or sweeteners and bone mass. Aspartame treatment has been shown to delay the onset of bone loss in rodents [31]; however the mechanism is likely to be independent of sweet taste receptors. In a related study we found that early exposure to saccharin increases bone mass later in life (unpublished data). From this observation, we might predict that T1R2 or T1R3 deficiency would result in a low bone mass phenotype. However, our studies *in vitro* indicate that effects of artificial sweeteners on adipocyte differentiation and metabolism are independent of T1R2 or T1R3 activation [16], suggesting that taste receptor KO models and artificial sweetener treatment models are not necessarily reciprocal systems. Further studies will be necessary to delineate taste receptor expression profiles in osteoblasts and osteoclasts, and to evaluate the effects of sweet taste receptor activation on bone development.

While there have been some reports of metabolic phenotypes in taste receptor KO animals, these studies have primarily been performed to characterize sweet taste receptor activity in the gut and pancreas. In such studies, T1R3 KO mice have impaired glucose tolerance in response to oral, but not intraperitoneal GTTs [14]. This result emphasizes taste receptor-stimulated GLP-1 secretion in the gut, rather than taste receptor-stimulated insulin secretion in the pancreas, as a significant driver of glucose homeostasis. However, an independent report shows that T1R2 expression is necessary for fructose-, but not glucose-stimulated insulin secretion from the pancreas [13]. Taken together, these results suggest that the sweet taste receptors of the gut and pancreas may have differential impacts on glucose homeostasis when presented with different ligands and/or diets. A similar paradigm may affect adipose tissue, in which the availability of an endogenous ligand may affect receptor activation and our interpretation of the role of sweet taste receptors. Further studies will be necessary to elucidate the identity of an endogenous ligand for sweet taste receptors *in vivo*.
